# Utilizing Biomass-Based Graphene Oxide–Polyaniline–Ag Electrodes in Microbial Fuel Cells to Boost Energy Generation and Heavy Metal Removal

**DOI:** 10.3390/polym14040845

**Published:** 2022-02-21

**Authors:** Asim Ali Yaqoob, Albert Serrà, Showkat Ahmad Bhawani, Mohamad Nasir Mohamad Ibrahim, Anish Khan, Hajer S. Alorfi, Abdullah M. Asiri, Mahmoud Ali Hussein, Imran Khan, Khalid Umar

**Affiliations:** 1 Materials Technology Research Group (MaTRec), School of Chemical Sciences, Universiti Sains Malaysia, Minden 11800, Penang, Malaysia; asim.sukhi@gmail.com (A.A.Y.); mnm@usm.my (M.N.M.I.); 2 Thin Films and Nanostructures Electrodeposition Group (GE-CPN), Department of Materials Science and Physical Chemistry, University of Barcelona, Martí i Franquès 1, E-08028 Barcelona, Catalonia, Spain; a.serra@ub.edu; 3 Institute of Nanoscience and Nanotechnology (IN2UB), Universitat de Barcelona, E-08028 Barcelona, Catalonia, Spain; 4 Faculty of Resource Science and Technology, Universiti Malaysia Sarawak (UNIMAS), Kota Samarahan 94300, Sarawak, Malaysia; sabhawani@gmail.com; 5 Center of Excellence for Advanced Materials Research, King Abdulaziz University, Jeddah 21589, Saudi Arabia; akrkhan@kau.edu.sa (A.K.); asiri2@gmail.com (A.M.A.); 6 Chemistry Department, Faculty of Science, King Abdulaziz University, Jeddah 21589, Saudi Arabia; hsalorfi@gmail.com (H.S.A.); mahussein74@yahoo.com (M.A.H.); 7 Applied Sciences and Humanities Section, University Polytechnic, Faculty of Engineering and Technology, Aligarh Muslim University, Aligarh 202002, India; imrannano@gmail.com

**Keywords:** microbial fuel cell, graphene oxide, electrodes, polyaniline, silver, wastewater treatment

## Abstract

Although regarded as environmentally stable, bioelectrochemical fuel cells or, microbial fuel cells (MFCs) continue to face challenges with sustaining electron transport. In response, we examined the performance of two graphene composite-based anode electrodes—graphene oxide (GO) and GO–polymer–metal oxide (GO–PANI–Ag)—prepared from biomass and used in MFCs. Over 7 days of operation, GO energy efficiency peaked at 1.022 mW/m^2^ and GO–PANI–Ag at 2.09 mW/m^2^. We also tested how well the MFCs could remove heavy metal ions from synthetic wastewater, a secondary application of MFCs that offers considerable benefits. Overall, GO–PANI–Ag had a higher removal rate than GO, with 78.10% removal of Pb(II) and 80.25% removal of Cd(II). Material characterizations, electrochemical testing, and microbial testing conducted to validate the anodes performance confirmed that using new materials as electrodes in MFCs can be an attractive approach to improve the electron transportation. When used with a natural organic substrate (e.g., sugar cane juice), they also present fewer challenges. We also optimized different parameters to confirm the efficiency of the MFCs under various operating conditions. Considering those results, we discuss some lingering challenges and potential possibilities for MFCs.

## 1. Introduction

Two of the greatest challenges that humankind currently confronts are the energy crisis and a scarcity of clean water. Recently, a fully developed prototype of a microbial fuel cell (MFC) was shown to generate bioelectricity while simultaneously removing pollutants from water [1,2,3]. This and other MFCs can also be used in methods in which bacterial species oxidize organic matter and produce electrons and protons [4]. MFCs differ from established energy cells in that they convert organic substrates directly into electric energy and operate efficiently in stable environments along with energy generation; they can also support wastewater treatment [5,6]. Comparison of MFCs with conventional energy storage devices is well explained in the previous literature [7,8,9,10,11]. Thus, given the ever-increasing global demand for energy and water, MFCs could prove to be an essential part of the solution [12]. Additional effort is needed to improve their performance in order to overcome difficulties with improving bacterial growth, facilitating electron transport, and developing cost-effective organic substrates. Each MFC contains two chambers: an anode chamber and a cathode chamber. The produced electrons use the outside circuit to travel from the anode electrode to the cathode electrode, whereas the protons move directly to the cathode with the help of a proton exchange membrane such as Nafion [13]. The proton exchange membrane cost is quite high, using a series of Nafion membranes, and membrane thicknesses influence the performance. Sun et al.’s research group provides a brief discussion on the Nafion membranes series in the literature [14]. However, within that dynamic, they mentioned the difficulty of poor electron transport stemming from the use of low-quality anode electrodes. To promote electron transfer and bacterial growth, low-cost, highly conductive anode electrodes are therefore required. Of all elements that directly impact bacterial development, the anode electrode is one of the few that may provide a steady environment free of toxicity. In the case of MFCs, the anode electrode is in direct contact with biofilm, thereby allowing the MFCs to generate and transport electrons more efficiently [15].

For electrodes, carbon-based materials have received considerable attention in electrochemistry due to their conductivity and surface area [16]. Although metal-based electrodes have demonstrated higher conductivity than carbonaceous electrodes, entire systems using them have shown weak long-term stability due to the corrosion of the metal-based electrode, which also compromises bacterial growth and activity [17]. By contrast, carbon-based materials have attracted attention as MFC electrodes, particularly in the form of carbon veils, rods, sheets, and foams. However, their energy performance has proven to be inadequate [18], and although carbon nanotubes and carbon black had greater conductivity than normal carbon, their toxicity in relation to the bacterial species have prevented their use in MFCs.

The toxicity towards microorganisms coupled with their vulnerability to oxidation have thus redirected attention to graphene derivatives [19]. Graphene derivatives are honeycomb-shaped, sp^2^-hybridized carbon atoms that outperform carbon nanotubes and other carbon materials in terms of thermal, mechanical, chemical, and electrical conductivity as well as exhibit good biocompatibility with bacterial activities. Nevertheless, using commercial graphene to construct anodes is extremely costly and limits the economic viability of MFCs using the material. Even so, employing the Hummers’s approach to synthesizing graphene oxide (GO) from biomass exemplifies a brilliant way to lower the cost of GO production, and GO anodes fabricated by using this approach have shown efficient energy performance, good biocompatibility, and affordability [20]. However, in GO’s structure, GO sheets possess several oxygenic functional groups, including the hydrophilic functional group responsible for the poor electrical conductivity of conjugated sp^2^-hybridized systems. In that light, GO’s electrical conductivity necessitates the use of a conductive polymer and metal nanoparticles to increase the material’s electrical conductivity and render it appropriate for use as an anode in MFCs [21,22].

To improve GO’s electrical conduction, conductive polymers such as polypyrrole, polylactic acid, and polyaniline (PANI) have been used as modifiers in fabricating anodes [23,24]. According to Thambidurai et al. [23], PANI is preferred to other conductive polymers for energy production due to its cost-effectiveness, excellent doping qualities, good conductivity, relative stability, and ease of manufacturing. For example, Yong et al. [25] have reported fabricating anodes with a PANI–graphene foam composite and achieving 768 mW/m^2^ energy efficiency in MFCs, or roughly 4 times the rate of graphene foam. PANI also improved the rate of electron transport and thus energy production. In addition, a PANI-based electrode with graphene ribbons used in MFCs has been reported by Zhao et al. [26], whose data revealed a sixfold increase in energy production compared with a bare electrode. Ibrahim et al. [27] also recently reported similar results using GO–PANI, and the stated power density was 0.107 mW/m^2^. They also added that they plan to employ metal oxide as a modifier to boost the material’s conductivity. In support, several recent studies have shown that introducing a metal oxide as a modifier facilitates major output in energy performance in MFCs [28,29,30]. As all of those results suggest, PANI has the potential to revolutionize MFCs.

Considering all of the above, we used silver (Ag) nanoparticles to fabricate a GO–PANI–Ag composite for use in fabricating anode electrodes. Because no research examining anodes in MFCs based on GO–PANI–Ag composites has been conducted, we focused on synthesizing GO from the trunk materials of oil palms, species of which abound in countries in the Association of Southeast Asian Nations (e.g., Thailand, Indonesia Malaysia, Vietnam, and Cambodia) [31]. With a scope confined to metal ions and laboratory-scale operations, we first fabricated the anode for MFCs with GO and a PANI–Ag modification, after which we conducted electrochemical testing to gauge its performance. We also assessed the effectiveness of MFCs with the anode based on the GO–PANI–Ag composite in decontaminating wastewater of Pb(II) and Cd(II). In the operation of the MFCs, local sugar cane juice was used as an organic substrate, which to our knowledge has never before been reported. Last, the performance of the fabricated anode under various environmental circumstances was validated with key parameters optimized (i.e., pH and organic substrate).

## 2. Method and Materials

### 2.1. Chemical Reagents

Our study involved using sugar cane juice from pasar Malam Gelugor, Penang Malaysia, as well as oil palm trunk material and oil palm trunk sap from the School of Chemical Sciences at the Universiti Sains Malaysia (11800 Minden, Penang, Malaysia), lead nitrate (R&M Chemicals), cadmium nitrate tetrahydrate (R&M Chemicals), polysulfones (PSFs) with approximately 35,000 M_w_ (Sigma-Aldrich), potassium permanganate (Sigma-Aldrich), ammonium persulfate (R&M Chemicals), sulfuric acid (95–97%, QRec), hydrochloric acid (99%, AR grade; QRec), sodium nitrate (Sigma-Aldrich), dichloromethane (90%; QRec), hydrogen peroxide (30–32%, QRec), glucose-modified filter paper (70 mm, Adventic), ethanol (approx. 95%, QRec), aniline (99.5%, Merck), silver nitrate (R&M Chemicals), phosphate buffer (pH = 7), and distilled water.

### 2.2. Preparation of Carbonized Carbon and Synthesis of GO

Following Shahriary et al.’s [32] approach, the oil palm trunk material was sliced into small pieces and heated in an oven at 1100 °C with an argon gas flow of 30 °C/min. To obtain carbonized carbon powder, the oil palm trunk material was next heated in a furnace for 3 h. Following carbonization, the carbon flakes were pulverized before being oxidized to synthesize the GO. Hummers’s technique was used to prepare GO from carbonized material because it is easier and less expensive than other approaches and produces a better-structured GO product without generating any harmful gases during synthesis [32]. The initial carbonized carbon powder (5 g) and sodium nitrate (6 g) were stirred in sulfuric acid for 1 h at 0–50 °C during the preparation reaction. After that, potassium permanganate (15 g) was added to sulfuric acid at less than 50 °C for 3 h, and an ice bath was used to keep the reaction temperature constant. The reaction mixture was subsequently stirred for 24 h without an ice bath to achieve full oxidation and violet–brown color. Next, while keeping the temperature at 90 °C, distilled water (150 mL) was slowly added to the mixture with continual stirring until turning dark brown. The reaction mixture was cooled to room temperature before water (150 mL) and hydrogen peroxide (30 mL) were added to reduce the influence of potassium permanganate. Last, the product was rinsed several times with distilled water and heated in a simple oven at 100 °C for 1 day to obtain the powdered form of GO, which was thereafter kept in airtight Teflon vials. The synthesized material was later used to make the GO–PANI–Ag composite and fabricate a GO anode.

### 2.3. Preparation of GO–PANI–Ag Composite

**Scheme 1:**
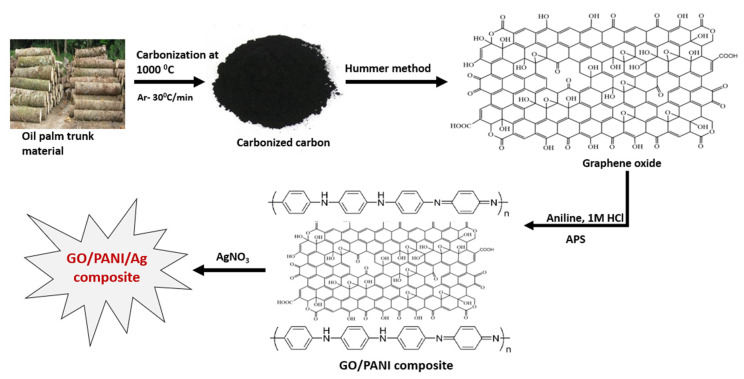
Conversion of palm trunk material into GO and, in turn, the GO–PANI–Ag composite (Modified from reference [27] with permission of MDPI).

To construct the GO–PANI–Ag composite, equimolar ammonium persulfate and aniline were mixed with GO solution (100 mL) to make a homogenous solution. The aniline was dispersed in hydrochloric acid (1 M), after which the reaction solution was sonicated for 60 min before being heated at 250 °C for 24 h. Afterwards, silver nitrate (5 g) was mixed with the GO–PANI solution and stirred for 24 h. To eliminate contaminants, the solid product of the GO–PANI–Ag composite obtained was washed with distilled water. The conversion of oil palm trunk material into the GO–PANI–Ag composite is depicted in Scheme 1. The similar preparation method of GO-PANI was also explained in previous work [21].

### 2.4. Material Characterization

The morphological and physiochemical properties of the synthesized material were analyzed with different techniques, namely thermal gravimetric analysis (TGA), transmission electron microscopy (TEM), X-ray diffraction (XRD), scanning electron microscopy (SEM), atomic force microscopy (AFM), and Fourier transform infrared (FTIR) spectroscopy.

### 2.5. Fabrication of the Anode Electrode

The anodes for MFCs were made using the GO obtained and the GO–PANI–Ag composite powder. Two different anodes were fabricated to assess their relative efficiency. The dried fine GO powder (5 g) was mixed with PSFs (2.5 g) to make a paste, which was then applied to the surface of a graphite rod (2 mm) as the sole current collector [24]. To make the solid cylindrical rod-shaped electrode, the coated GO material on the graphite rod was heated at 50 °C for 12 h. To make a GO–PANI–Ag composite anode, the GO–PANI–Ag composite (5 g) was also mixed with PSFs (2.5 g) to make a paste, which was then similarly deposited on a graphite rod. The conductivity and other functions of each electrode were determined according to its surface material. In both anodes, the component ratio, size of the electrode (7.51 × 7.51 cm), and its surface area (0.0050 m^2^) were identical.

### 2.6. Operation of MFCs

#### 2.6.1. Inoculation Source

Local wastewater was collected from Bayan Lepas, Malaysia, and treated with 100 parts per million of lead and cadmium ions to become synthetic wastewater. The physiochemical properties of the untreated wastewater and synthetic wastewater were investigated, the results of which appear in Table 1. Their conductivity, pH, and temperature were measured using an electrical meter (Alpha-800 conductivity meter), pH meter (instrument 700, Eutech, Paisley, UK), and a thermometer (GH, ZEAL Ltd., London, UK), respectively.

#### 2.6.2. Configuration and Operation of MFCs

We used MFCs with two chambers separated by Nafion proton exchange membrane, each with a length and diameter of 10.0 × 9.5 cm, a total volume of roughly 1000 mL, but an operating capacity of only 500 mL. The anode chamber of the MFCs had synthetic wastewater (500 mL), while the cathode chamber had tap water with phosphate buffer (0.1 M), used to maintain the pH of the source of anode inoculation. The pH of the anode was 6.45 and of the cathode was 7.0. The anode chamber was sequestered from the outside environment, whereas the cathode chamber had an external oxygen source using an aquarium pump. Although the anode chamber was sealed, 5 mL sugar cane juice was given to the bacteria every day to boost their activity. The wastewater contained a mixed bacterial culture that generated bioenergy and removed metal ions by oxidizing the organic substrate (i.e., sugar cane juice). As the fabricated anode electrode was employed in each reaction, the cathode electrode was a graphite rod similar in size to the anode electrode, and they were separated by a distance of 7 cm. In the presence of external resistance (1000 Ω), electrons were transferred from the anode to cathode using a copper (Cu) electric wire. Both MFCs tests with the GO and GO–PANI–Ag anodes were performed in the same manner and at room temperature for 35 days. 

### 2.7. Electrochemical Tests

A digital multimeter was employed to measure the voltage trend (UNI-T, Model 33B^+^, China). Ohm’s law was used to determine the power density (PD) and current density (CD), which along with internal resistance (r) were calculated using Equations (1)–(4):

(1) V=IR

(2) $$\mathrm{PD}=\frac{V^{2}}{\mathrm{RA}}$$

(3) $$\mathrm{CD}=\frac{I}{A}$$

(4) $$r=\left(\frac{E-V}{V}\right)R$$

in which I is the current, V the voltage output, A the cross-sectional area, r the internal resistance, R the external resistance, and E the electromotive force, calculated by measuring the voltage of an open circuit using a voltmeter with a high-resistance connection. Polarization behavior, generally requiring a 30-min variation period to evaluate, was observed using the so-called “REXT variation” method, which involves using different resistances of 5000, 4000, 3000, 2000, 1000, 500, 300, 200, and 100 Ω. After the reaction reached a pseudo-steady state, the polarization curve was examined. Using cyclic voltammetry (CV), the redox reaction during the operation was investigated (Model BAS Epsilon Version 1.4; West Lafayette, IN, USA). On Days 1 and 35 of the reaction, a cyclic voltammogram was recorded using a potentiostat device with a scan rate of 5 mV/s within the potential range of +0.8 to −0.8 V. In-situ electrochemical tests were performed. In addition, to complete the CV evaluation, the specific capacitance (C_p_, (F/g)) was also calculated. Equation (5) was used to calculate the value of C_p_.

(5) $$C_{p}=\frac{A}{2\mathrm{mk}\left(V2-V1\right)}$$

where $\left(V2-V1\right)$ = potential range of CV; m = loaded sample (g), A = area of CV curves, k = scan rate of CV in mV/s.

### 2.8. Removal Efficiency and Anode Morphology

Regarding the removal of pollutants from wastewater as another significant application of MFCs, the produced anode showed improved removal efficiency by enabling bacterial activities [9]. An atomic adsorption spectrometer (AAS) (A Analyst 400, PerkinElmer) was used to analyze the metals concentration. Every 5 days, a sample (1 mL) was taken from the anode chamber for AAS analysis, and efficiency was estimated by interpreting AAS values using Equation (6):

(6) $$\mathrm{RE}\;\%\;=\frac{Ti-Tf}{Ti}\times 100$$

in which RE is the removal efficiency, T*i* is the initial concentration, and T*f* is the final concentration. The growth and development of the biofilm were responsible for the removal efficiency and bioenergy generation of the MFC. Next, SEM was used to investigate the biofilm stability and growth, and SEM images revealed a stable biofilm on the anode. Bacterial growth on the anode’s surface, confirmed with SEM images, indicated that the anode is biocompatible with live microorganisms, as further discussed in Section 3.

### 2.9. Bacterial Identification from Anode Surface

The biofilm (1.00 mm) was scraped from the anode’s surface and preserved in distilled water after the operation of the MFCs in order to identify the bacterial species. The colonies were transferred to nutrient agar plates using serial dilution. After some time, distinct colonies appeared on the plates, which were purified and studied carefully to determine the bacterial species. The pure cultured nutrient agar plates were stored in the refrigerator until measurement was completed. Next, a polymerase chain reaction was used to identify the microbial 16S rRNA genes. To enhance that process, a forward primer (27F) and reverse primer (1492R) were utilized. The product amplified by the polymerase chain reaction was cloned using a cloning kit (TOPO TA, Carlsbad, Invitrogen, Waltham, MA, USA). Last, 16S rRNA bacterial species were collected from GenBank.

### 2.10. Different Parameter Optimization

Using the GO and GO–PANI–Ag composite anodes, we studied only two parameters, pH, and organic substrate, given their direct impact on the performance of MFCs. The parameters for GO and GO–PANI–Ag composite anodes generally need to be optimized because they directly affect electron transport and bacterial growth. For both the GO and GO–PANI–Ag composite anodes, many experimental trials were performed to optimize the findings. To adjust the pH to the optimal range of 4–10, HCL and NaOH were used, and after 10 days, each range (i.e., energy and removal efficiency) was recorded. All other variables (i.e., external load, temperature, and electrodes) were identical. Commercial glucose and sap from local oil palm trunks were utilized for comparison to confirm the sugar cane juice’s excellence as an efficient organic source of sugar. Other parameters (i.e., pH = 7, room temperature, and 1000 Ω external resistance) were kept constant across the trials, and the measurements of energy and removal efficiency were recorded after 10 days. We used the same inoculation source for bacterial activity during our optimization of parameters.

## 3. Results and Discussion

### 3.1. Material Characterizations

#### 3.1.1. FTIR Analysis

**Figure 1:**
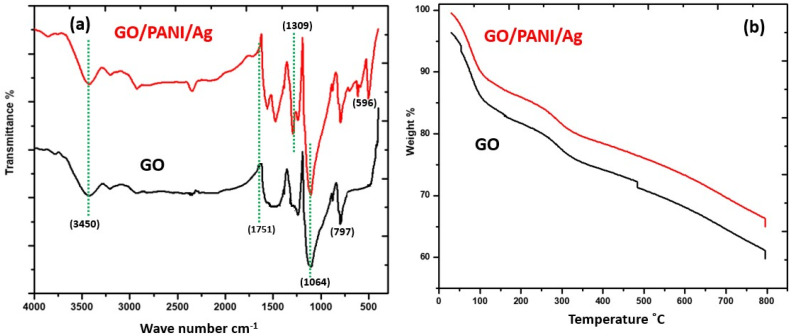
(**a**) FTIR spectroscopy and (**b**) TGA of the synthesized biomass-based GO and GO–PANI–Ag composite.

As shown in Figure 1a, the FTIR spectra of the GO and GO–PANI–Ag composite were investigated in the 500–4000 cm^−1^ range. The existence of oxygen in the product was confirmed by the appearance of a –OH group peak at 3450 cm^−1^ in the GO spectra. Furthermore, GO spectra at 1751, 150, 1064, and 1309 cm^−1^ revealed the presence of C=O, C=C, and C–O [33]. In the GO–PANI–Ag composite, however, a band at 2400 cm^−1^ appeared due to the reshuffling of the structural arrangement after the composite was formed. A peak at 596 cm^−1^ indicated the presence of Ag in the composite, while the 1064 cm^−1^ band was linked to electron delocalization due to C–N vibrational stretching in the quinoid structure. The latter property suggests that the product included PANI doped with the GO structure. Beyond that, the band at 797 cm^−1^ corresponded to C–H deformation bending in the benzene ring. The PANI and Ag absorption bands are correlated to those types of absorption bands [34]. Although the peaks of the GO–PANI–Ag composite were entirely red- shifted owing to the interaction between GO, PANI, and Ag, there was no particular difference band [35]. The position of the Ag metal in the IR region was observed to be between 600 cm^−1^ and 3400 cm^−1^ [36]. The polaron-polaron interaction between the planer polyaniline and silver metal was noticed, which caused shifting owing to the bulky nature of the polyaniline for Ag, as well as ring deformation in the aniline ring. Out of plane bending, a deformation for the C-H bond, is present in functionalized benzene, as evidenced by the presence of a peak in the IR spectrum at 797 cm^−1^. Due to the metal in that region, the same peak was also getting broadening [37]. 

#### 3.1.2. TGA Analysis

TGA analysis was used to assess the thermal stability of the produced GO and the GO–PANI–Ag composite, as shown in Figure 1b. Compared with the GO–PANI–Ag composite, GO displayed low stability. At 800 °C, for instance, the GO–PANI–Ag composite had 67% remaining mass ratio of the composite, whereas GO had 57%. According to the trend, the decreasing curve in weight was larger for GO than for the composite. The thermal decomposition of the –COOH, –OH, and –CO functional groups may have caused significant loss in GO at approximately 200 °C. A similar loss was detected in the GO–PANI–Ag composite at 150 °C, which indicated the loss of water molecules, whereas a larger loss was found above 300 °C that indicated the disintegration of PANI–Ag. The produced material thus seems ideal as an electrode, for it can operate more efficiently up to the maximum operating temperature for MFCs [34].

#### 3.1.3. XRD Analysis

**Figure 2:**
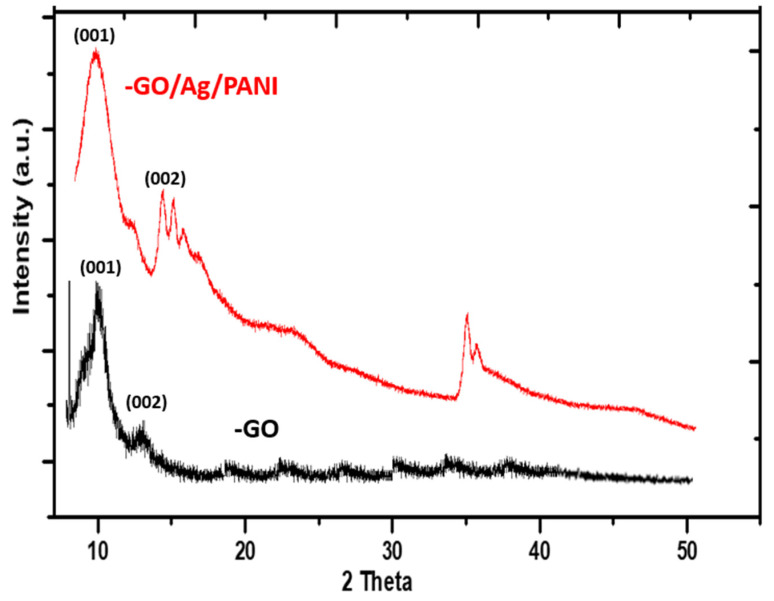
XRD spectra of biomass-based GO and GO–PANI–Ag composite.

The XRD spectra of the GO and GO–PANI–Ag composite are presented in Figure 2. The peak at 10.2° in the GO’s XRD spectra was linked to the efficient oxidation of carbon. As stated in the literature, the peak at 001 indicated the synthesis of GO. At 10.2°, the final brownish-black color peaked. Another peak arose at 002, which was thought to correlate to graphite oxidation [34]. GO has abundant interlayer space containing oxygenated functional groups, as seen by the peak moving from 002 to 001. In another example, peaks at 15.1°, 19.25°, 25.6°, and 38.0° matched the peaks of PANI–Ag, as Yan et al. [38] have previously observed. The existence of a lengthy chain of PANI with a well-ordered structure was revealed by the peak at 2θ = 6.5°. The two peaks in the literature also present in the XRD spectra of the GO–PANI–Ag composite convincingly supported the existence of PANI–Ag in the composite. The GO–PANI–Ag composite had a strong peak at 19.25°, with an interspacing of 3.4 A° that indicated its greater crystallinity than GO. This finding revealed that adding PANI enhanced the material’s crystalline properties, possibly due to the efficient polymerization of aniline.

#### 3.1.4. SEM and TEM Analysis

**Figure 3:**
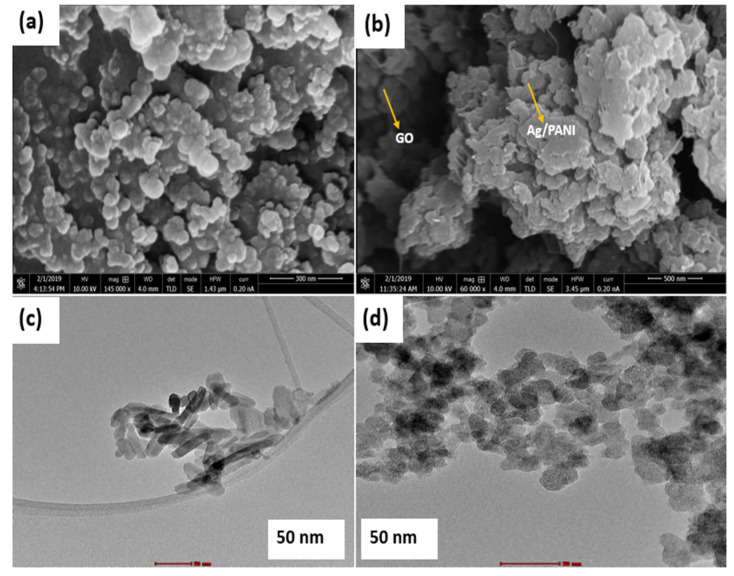
SEM images of (**a**) biomass-based GO and (**b**) the GO–PANI–Ag composite and TEM images of (**c**) the biomass-based GO and (**d**) the GO–PANI–Ag composite.

The morphology of GO and the GO–PANI–Ag composite was studied using SEM imaging. In line with previous reports [39,40,41,42], GO SEM image (Figure 3a) revealed an uncomplicated particle shape that was not uniform on the surface. This type of particle has a size of less than 100 nanometers [39]. The fibrous shape of the GO–PANI–Ag composite was also visible via SEM (Figure 3b), as was the presence of PANI–Ag on the GO sheet, which appeared to be crystalline rod in structure. SEM additionally revealed that the GO–PANI–Ag composite was successfully formed. As illustrated in Figure 3a, the PANI fibers appeared to be randomly scattered between or on the surface of the GO sheet. However, as shown by XRD spectra, PANI–Ag had a highly organized, compact structure due to the tight bonding between GO and PANI–Ag, which can be predicted to improve electron transport in MFCs [40,41].

TEM was also used to determine the GO’s structure, which included many wrinkles and scrolls, as shown in Figure 3c. TEM images of GO revealed a high level of transparency indicating effective carbon oxidation. Because the conjugated GO structure broke during oxidation, the GO’s structure displayed a thin film shape with characteristic compactness. Figure 3d shows that the composite material had a slightly tubular shape, thereby suggesting the presence of PANI. There were also some flaky structures indicating that the polymerization of aniline and the creation of the composite with the GO sheet had succeeded [42]. The effective fabrication of the composite electrode was thus predicted to improve the movement of electrons.

#### 3.1.5. AFM Analysis

**Figure 4:**
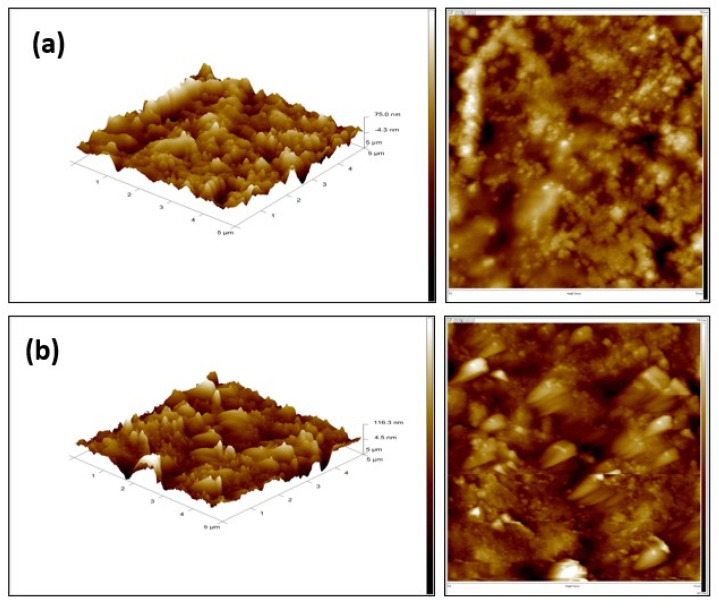
AFM analysis of (**a**) GO and (**b**) the GO–PANI–Ag composite.

Figure 4 shows the results of our AFM analysis of GO and the GO–PANI–Ag composite, with the fine, smooth surface of the processed material shown in 2D and 3D. The GO layers were arranged asymmetrically, as shown in Figure 4a, whereas the surface of the GO–PANI–Ag composite was smooth and even, as shown in Figure 4b. The particle arrangement in the composite was highly organized and regular, which suggests greater biocompatibility for bacterial growth. The synthesized material was also ordered well and had a compact structure and surface according to AFM scans. Using the material as an anode for the operation of MFCs thus seems favorable, and structural and morphological characterizations of the synthesized material indicated its suitability as a raw material for producing anode electrodes.

### 3.2. Electrochemical Trials Using the Fabricated Electrodes

#### Power Generation and Polarization

**Figure 5:**
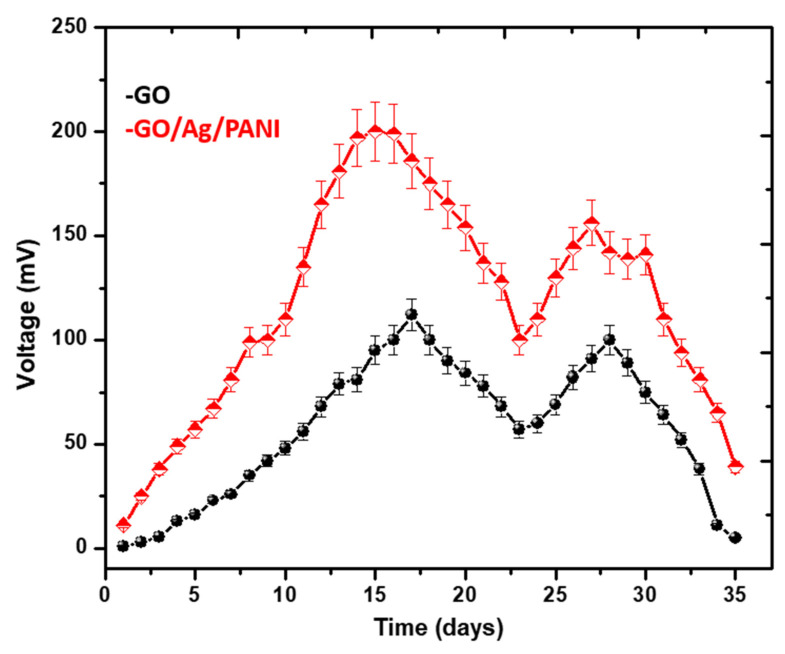
Power generation trend of the GO and GO–PANI–Ag composite anodes in operation in MFCs over 35 d.

To examine the voltage generation trends of the anodes, the fabricated GO and GO–PANI–Ag composite were employed in MFCs in experiments that each took 35 days to complete. A significant difference in voltage production emerged in the early days of operation, with GO delivering 1 mV and the GO–PANI–Ag composite anode delivering 11 mV. In time, the voltage production of GO and the GO–PANI–Ag composite began to differ significantly, and on Day 10, for example, GO generated 48 mV and the GO–PANI–Ag composite 110 mV. On day 17, the GO generated voltage was 112 mV (current = 0.112 mA), whereas the GO–PANI–Ag composite generated 199 mV (current = 0.199 mA) on day 16. The following day, the GO–PANI–Ag composite generated 186 mV and thus more than GO. In both cases, the voltage trend increased steadily; however, the GO–PANI–Ag composite generated far more voltage than the unmodified GO. The organic substrate provided was gradually oxidized by the bacterial colony in the beginning, and the voltage progressively increased. The results of the GO–PANI–Ag composite indicated that adding PANI–Ag enhanced electron transport and was biocompatible given its lack of toxicity toward live bacteria. Both electrodes were in operation until the voltage generation ended. After 18 days, both electrodes demonstrated a downward trend in voltage production, and by day 35, the voltage was very poor in both cases. Such steady growth was caused by the initial fresh growth of bacterial species in the anode chamber, when the bacteria quickly began oxidation and electron transport was high. The newly fabricated anode facilitated the rapid transport of electrons produced by bacteria during the oxidation of the organic substrate. Voltage later decreased because in their life cycle, bacterial species eventually reach the death phase, and both electrodes exhibited less performance at a certain point. That outcome suggests that the bacterial species was good only for 35 day of maximum voltage output before low oxidation, at which point electron generation was low. According to earlier literature, one of the causes for the voltage trend decreasing throughout operation is the use of organic substrate. The organic substrate must be sufficient to supply fuel for bacterial activities for an extended survival [43,44]. In the present study, both electrodes showed excellent performance because electrodes are used to transport the electrons. The generation of electrons without decreasing trend during operation is dependent on bacterial activities and organic substrate stability [45]. Figure 5 shows that the voltage trend steadily grew and then declined until the day 35 of the MFCs was complete.

**Figure 6:**
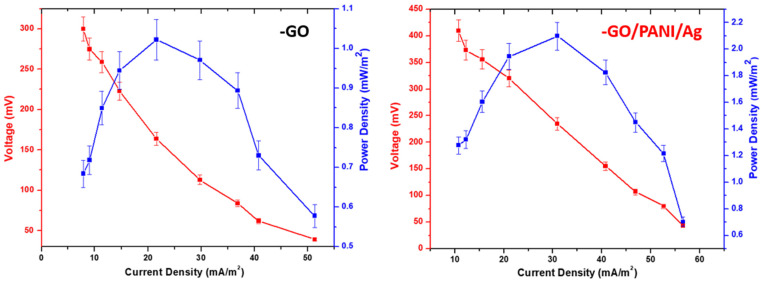
Polarization behavior of the GO and GO–PANI–Ag composite anodes.

We also investigated the polarization behavior of both materials in order to clarify the relationship between current and resistance in MFCs. In our analysis of open circuit voltage, external resistance was changed from 5 kΩ to 100 Ω as a means to study the polarization curves and determine the optimal resistance–current relationship. Polarization behavior was investigated on day 17 of the operation, when the voltage trend in both cases was high. High external resistance indicated limited voltage generation but swift voltage stabilization, whereas low external resistance indicated high voltage generation but inexact voltage stability. The maximum CD was 51.31 mA/m^2^ at 100 Ω external resistance in the GO anode, while the highest PD was 1.022 mW/m^2^. In this anode, the internal resistance of the solution was 1134 Ω. From higher to lower external resistance, the GO indicated an increasing tendency in CD; its high external resistance, for example, coincided with 7.89 mA/m^2^ at 5 Ωk, 21.57 mA/m^2^ at 1 Ωk, and 51.31 mA/m^2^ at 100 Ω. Similarly, the CD of the GO–PANI–Ag composite anode (56.57 mA/m^2^) was high at 100 Ω external resistance with an internal resistance effect of 810 Ω. For the composite anode, the maximum PD was 2.09 mW/m^2^, as shown in Figure 6. The electrons could transfer from the anode, which had lower energy efficiency due to high external resistance. Furthermore, the internal resistance was reduced due to the obstructed passage of electrons from the bacteria to the anode’s surface. A similar pattern in polarization experiments has previously been discussed [46,47,48]. 

**Figure 7:**
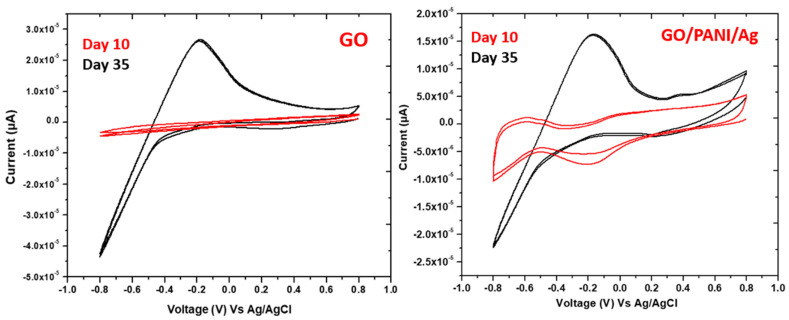
CV curves of GO and the GO–PANI–Ag composite anodes during the operation of MFCs.

We next examined the electron transport rate using in-situ CV performed at a rate of 5 mV/s in the potential range of +0.8 to −0.8 V in PBS (pH = 7) as an electrolyte solution. The bacterial biomass catalytic activity at the electrodes was proven by the CV analysis at different intervals, which was reflected in the redox reactions that happened throughout the operation. Figure 7 shows the CV behavior of both anodes during the operation of MFCs at various intervals. Both the forward scan (FS) and reverse scan (RS) were taken of GO’s CV curves; the FS was −0.3 × 10.0^−5^ µA on day 10 and 2.5 × 10.0^−5^ µA on day 35, whereas the RS on days 10 and 35 was −0.3 × 10.0^−5^ µA and −4.5 × 10.0^−5^ µA. The FS and RS were also measured for the GO–PANI–Ag composite on days 10 and 35; the results for FS were 2.5 × 10.0^−6^ µA and 1.5 × 10.0^−5^ µA on those days, respectively, whereas the results for RS were −2.2 × 10.0^−5^ µA on both days. For GO, the maximum FS was 2.5 × 10.0^−5^ µA, while the lowest RS was −0.3 × 10.0^−5^ µA. However, for the GO–PANI–Ag composite, the maximum FS was 1.5 × 10.0^−5^ µA and the highest RS −2.2 × 10^−5^ µA. The oxidation and reduction rates at different periods of the operation of the MFCs can be represented by the FS or RS. On day 35, the oxidation peaks of GO and the GO–PANI–Ag composite were high, and the greatest rate of decrease related to reverse electron transport on that day. The oxygen supply to the cathode chamber enhanced the electron discharge rate and helped to neutralize electrons before moving to the anode on day 35, thereby resulting in maximal oxidation and reduction. The biological process in the GO took longer than in the GO–PANI–Ag composite because there were no visible oxidation or reduction currents on the 10th day, which corresponded to the low metal removal at this time. Considering that the standard reduction potential for Cd(II) is −0.403 V and for Pb(II) is −0.126 V (at 25 °C), these values may be used to infer from the CV curves. A peak fall in CV will be shown if a metal reduction has occurred. In the case of the GO, reduction maxima at −0.8 V were found on the 10th and 35th days, respectively, at a maximum reduction current of −0.3 × 10.0^−5^ µA and −4.5 × 10.0^−5^ µA. This might indicate that additional species were formed during the fermentation of organic materials, leading the system to have a higher reduction potential and the metals to precipitate before the tests. By the 10th day, for example, 24.01 % of the metal (Pb(II)) was removed, and on the 35th day, more than 50 % of the metals had been removed. (Refer to Table 2). The maximum oxidation was 1.5 × 10.0^−5^ µA and the maximum reduction was −2.2 × 10^−5^ µA in the case of the GO–PANI–Ag composite. In this situation, the largest reduction peaks at −0.8 V (35th days) may correlate to the reduction of Cd (II) among the other chemicals in the system, including Pb(II), under these circumstances. The proportion of metals removed for the days the measurements were collected agrees with the CV curves in all three situations (see Table 2). Other parameters were also considered while evaluating the biofilm development trend at the GO and GO-PANI-Ag anodes during reaction. C_p_ may be calculated using the CV curve and Equation (5). The observed C_p_ indicated that the biofilm was quite strong during the reaction. High C_p_ values suggested that the biofilm was stable and formed well. The GO shows 7 × 10^−5^ on day 35 while GO-PANI-Ag shows 5 × 10^−4^ on day 35. Similar findings have been observed in other research to explain and imply significant biofilm development. Similar patterns have also previously been reported for other materials [41,49].

### 3.3. Removal of Pb (II) and Cd (II)

The most recent strategy for extending the use of MFCs is using them to remove inorganic pollutants from water. Because the presence of Pb(II) and Cd(II) ions in water resources can result in diseases that are potentially fatal to humans [50], we sought to employ MFCs to remove the majority of hazardous metal ions, namely Pb(II) and Cd(II), from synthetic wastewater. Sugar cane juice provided energy for the bacterial species to grow around the electrodes, while the biofilm that formed on the anode surface was completely responsible for the efficiency of removal. Because the fabricated materials had abundant biofilm surrounding the surface of the anodes, as shown in Table 2, the effectiveness of removal was outstanding. When compared with unmodified GO, the GO–PANI–Ag composite anode was highly effective in remediating Pb(II), at a rate of 78.10% versus 57.52%. Similar results emerged for the remediation of Cd(II), with the GO–PANI–Ag composite achieving 80.25% remediation and GO achieving only 64.00%. In both cases, the removal efficiency of the GO–PANI–Ag composite improved. Compared with unmodified GO, the introduction of PANI–Ag showed enhanced biocompatibility for the development of bacterial communities. Overall, both electrodes were biocompatible enough for bacterial growth and respiration; although the efficiency of removal was weaker than in earlier research, the organic substrate provided eventually could no longer offer enough energy for bacteria to perform respiration. The lack of organic substrate also caused the bacterial species to enter the death phase, which prevented the metal ions from being converted into an insoluble state. In past work, Singh et al. [51] investigated the effects of various organic substrates and determined that the lack of an organic substrate supply could lower the efficiency of MFCs. It can thus be inferred that the organic substrate employed is adequate for bacterial activities in terms of removing metal ions but that more effort is required for high energy generation. 

**Figure 8:**
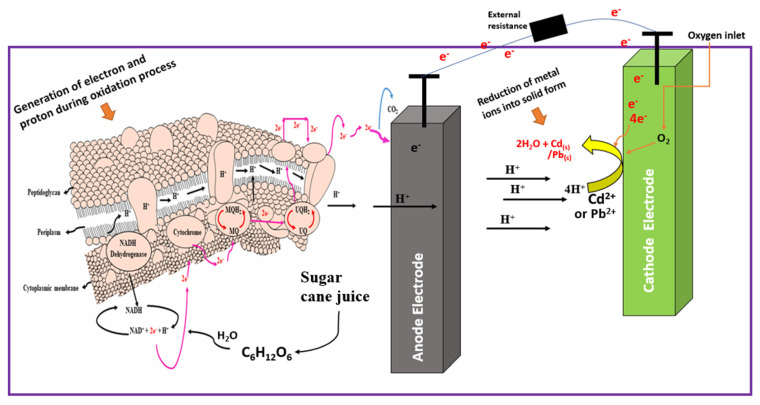
Mechanism of MFCs in the generation of electrons and the reduction of metal ions (Reproduced from reference [53,55] with Elsevier and Springer nature permission).

AAS is only used to observe metal concentrations, as we described earlier. According to prior research, soluble metal ions in MFCs become insoluble (sludge form) after the MFCs’ operation. In our previous work, the MFC mechanisms (electron generation and transportation) are well explained [20,52,53]. Furthermore, the reduction process occurred when the electrons were effectively delivered to the cathode through the outside circuit. Through an electrochemical reduction process, the soluble form of metal ions (Pb and Cd) accept the generated electrons and were transformed into solid forms (precipitates) [54]. We also received a sludge at the end of operation. Figure 8 shows a systematic picture of the metal ion transformations. The reduction reactions for metals can be written as follows:

- Conversion of Cd^2+^ to Cd_(s)_
  Cd^2+^ + 2e^−^            Cd_(s)_
  2Cd^2+^ + 2H_2_O            2CdO + 4H^+^
  CdO + 2e^−^ + 2H^+^            Cd_(s)_ + H_2_O
- Conversion of Pb^2+^ to Pb_(s)_
  Pb^2+^ + 2e^−^            Pb_(s)_
  2Pb^2+^ + 2H_2_O            2PbO + 4H^+^
  PbO + 2e^−^ + 2H^+^            Pb_(s)_ + H_2_O

#### Characterization of Biofilm and Identification of Bacteria

**Figure 9:**
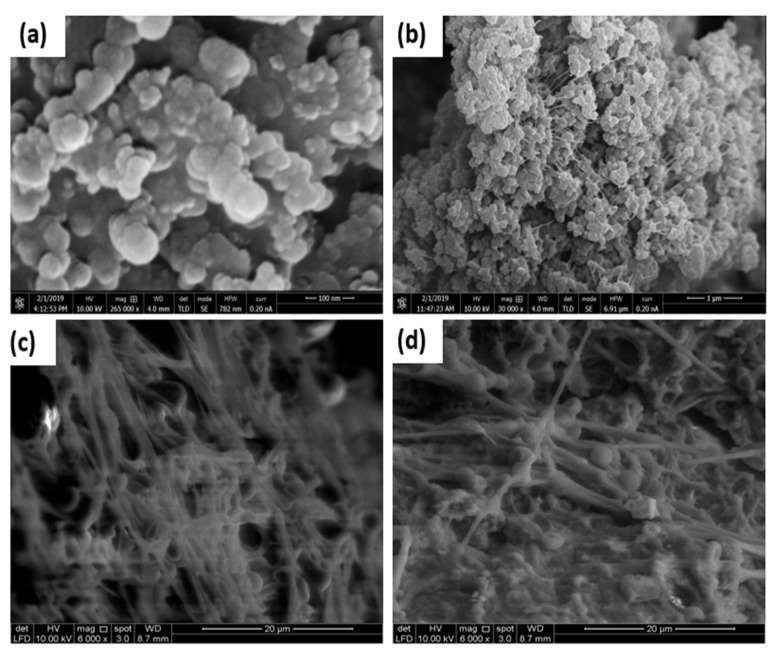
SEM images before and after the operation of MFCs: (**a**) GO anode before operation, (**b**) GO–PANI–Ag anode before operation, (**c**) GO anode after operation, and (**d**) GO–PANI–Ag anode after operation.

The strength and quality of biofilms directly impact the energy production and removal of metal ions achievable by anodes. To be clear, *biofilm* is a bacterial layer that grows on the surface of anodes, and because it occurs naturally during the operation of MFCs, its formation does not necessitate any particular treatment. A whopping 97% of biofilm is water, with 2–5% bacterial cells, 3–6% extracellular polymeric substances (EPSs), and trace amounts of mineral ions [51]. A variable that keeps bacteria on the anode surface for electron production is EPSs, comprised of nucleic acids (10%), polysaccharides (40–95%), lipids (40%), and proteins (1–60%) [44,56,57]. EPSs are also a hydrated component of biofilms that retain 97% of their water content via hydrogen bonding. The amount of EPSs in a given biofilm greatly increases the biofilm’s age; however, when the organic substrate becomes scarce, EPSs will begin to decline, thereby reducing the efficiency of MFC activities. To achieve excellent performance with MFCs, their biofilms have to be healthy and grow efficiently. The biofilms formed on the surface of the prepared GO and GO–PANI–Ag composite anodes were examined using SEM, and the fabricated anodes were tested before and after the procedure, as shown in Figure 9a–d, which clearly show the thick development of biofilm surrounding the anode’s surface. The mixed bacterial culture formed a biofilm layer around both surfaces, thereby demonstrating that the anodes are highly biocompatible for bacteria and have no harmful effects. Although both electrodes revealed a mixed colony of bacteria, SEM images revealed filamentous appendages.

The conductive pili on the filamentous appendages aid the transmission of electrons from bacterial cells to anodes. In previous research [20], we have detailed the electron transport pathways and shown how anode–cell contact is critical for high-performing MFCs. In our study presented here, we also discovered that a mixed culture of bacteria, the most prominent of which appear in Table 3, was accessible throughout the period of bacterial identification. Both anodes displayed essentially the same bacteria species, including *Leucobacter* spp., *Pseudarthrobacter* and *Arthrobacter* spp., and *Canibacter oris*, due to having the same water source. The presence of a mixed culture is a positive indicator of effective energy generation and the effective removal of heavy metals, which rank as some of the most dangerous pollutants in any environment. According to prior research, the identified species shown in Table 3 can both generate energy and successfully remove heavy metals. For example, Sturm et al. [58] investigated the effect of *Leucobacter* spp. and found that they had highly metal-resistant strains. In our study, *Leucobacter* spp. on the anode surface significantly lowered metal tolerance and thus demonstrated its potential for remediating Pb, Cd, and Cr, all of which are harmful environmental pollutants. By contrast, we also found that *Leucobacter*, *Pseudarthrobacter,* and *Arthrobacter* spp. served as active biocatalysts in generating energy and removing the Pb(II) and Cd(II) ions from wastewater.

### 3.4. Parameter Optimization

Optimizing multiple parameters was critical for determining whether our innovative electrode and organic substrate could be employed in the operation of MFCs. Using GO and GO–PANI–Ag anodes with 100-Ω resistance, we investigated several parameters—pH and organic substrate—while using synthetic wastewater containing the metal ions Cd(II) and Pb(II) as the inoculation source.

#### 3.4.1. Effect of pH on the Operation of MFCs

**Figure 10:**
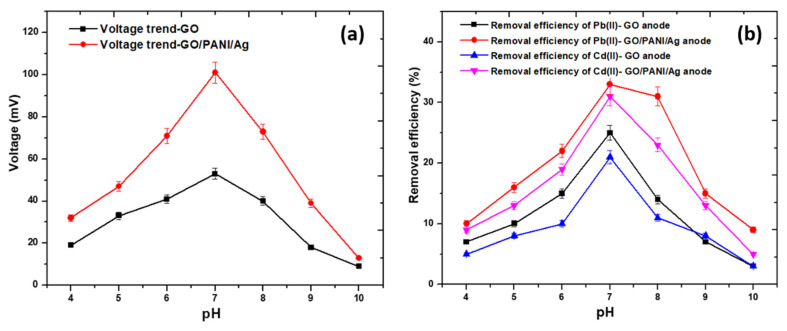
The effect of pH on (**a**) voltage trend and (**b**) the efficiency of metal removal while using the GO and GO–PANI–Ag anodes in different pH ranges.

Because living organisms cannot exist in acidic or alkaline environments without intervention, the pH of synthetic wastewater immediately affects how efficiently metal ions in MFCs produce energy and remove contaminants. Figure 10a shows that at a pH of 7, instead of under severely basic or acidic conditions, energy generation was high. As in other studies, we utilized buffer reagents to keep the solution pH within acceptable limits. Our observations revealed that pH of 4, 5, 9, and 10 did not provide favorable circumstances for the nutritious formation of biofilms on the anode surface. For example, respective voltages of 19 mV and 32 mV were reached with GO and GO–PANI–Ag at a pH of 4, whereas respective voltages of 9 mV and 13 mV were achieved at a pH of 10. Compared with severely acidic or alkaline conditions, a pH of 7 provided voltages of 53 mV with GO and of 101 mV with GO–PANI after a 10-d operation, and those values were higher than the ones observed at pH of 4, 5, 6, 8, 9, and 10. In addition, some energy output was attained in both acidic (pH = 4) and basic (pH = 10) conditions. At the same time, pH also impacted the efficiency with which Pb(II) and Cd(II) were removed from synthetic solutions. The neutral pH (pH = 7) provided excellent removal efficiency, for example. After the 10-day operation of the MFCs, GO removed Cd(II) and Pb(II) at rates of 21% and 25%, respectively, whereas GO–PANI–Ag removed them at respective rates of 31% and 33%. As the pH affected the biofilm’s activities, the bacterial activity was affected as well, which resulted in poor removal efficiency and energy generation [59]. In past research, Huang et al. [42] investigated pH influence on energy performance and the efficiency of removing Cd(II) using MFCs and concluded that the neutral pH can produce high amounts of energy compared with pH of 2–5 or even a pH of 9. Due to the disruption of biofilm, however, they likewise demonstrated 0% removal efficiency at pH of 2 and 10. In other work, Yuan et al. [60] described the influence of pH on the development of biofilm, which is responsible for removal efficacy, and observed that a pH of 5 is not preferred to a pH of 9, the latter of which allowed 2.5 times more energy efficiency than a pH of 5. In our study, pH of 5 and 6 facilitated higher energy and removal efficiency than pH of 9 and 10. After 10 days of operation, readings in each range were made, as shown in Figure 10a,b.

#### 3.4.2. Assessment of Organic Substrates in the Performance of MFCs

The organic substrate is a significant factor to consider while optimizing the performance of MFCs, and several studies have shown unique results using various natural organic substrates [42]. Any carbohydrate source used as an organic substrate for the operation of MFCs can provide energy to bacteria for their oxidative respiration activity [61]. In prior research, however, there have been very few attempts to use natural organic substrates in MFCs such as biomass waste, vegetable cellulose, brewery wastewater, mangroves, and wastewater from the chocolate industry. Among them, Salvin et al. [62] employed mangroves in MFCs as an organic substrate to improve energy performance. Local sugar cane juice, by contrast, was employed as an organic substrate in our investigation, along with commercial glucose and natural Malaysian oil palm trunk sap, to test the relative performance of the sugar cane juice. Although these carbohydrate-based organic substrates include starch, fibers, and simple sugar, oil palm trunk sap has more bacterial nutrients than sugar cane juice. The performance of all organic substrates is captured in Table 4, which shows that oil palm trunk sap performed the best. According to earlier research, more than 70% of nutrients are bacteria-friendly and thus allow bacteria to thrive and respire more efficiently [63]. 

## 4. Concluding Remarks and Recommendations for Future Research

This paper highlights the efficiency of MFCs, which remove extremely hazardous metal ions from synthetic wastewater while also generating electricity. MFCs rank among the most recent configurations for bioelectrochemical fuel cells and are more environmentally friendly, cost-effective, and readily controlled than their alternatives. Our work has demonstrated that a modified graphene–PANI–Ag anode fabricated from oil palm trunk material increased electron transport and thus achieved excellent energy performance. The natural sugar cane juice utilized in the MFCs also served as an organic substrate for bacterial growth and activity.

In the limited research on the use of natural organic substrates, different approaches have been used to assess the efficacy of energy generation and wastewater treatment while using MFCs. In our study, the fabricated electrodes were exceptionally stable and biocompatible for the operation of MFCs according to various material characterizations and findings regarding MFC operations. The presence of a solid biofilm around the anodes demonstrated that the GO and GO–PANI–Ag anodes were nontoxic and chemically stable for up to 35 days. Furthermore, two toxic metals, Pb(II) and Cd(II), were removed, and substantial results were achieved in a relatively short period. The MFCs were operated with two electrodes, GO and GO–PANI–Ag, using sugar cane as an organic substrate. Each MFC procedure required 35 days to complete and only 17 days to achieve the maximum energy efficiency. The GO achieved 51.31 mA/m^2^ and the GO–PANI–Ag 56.57 mA/m^2^ within 17 d of operation. The PD of GO was 1.022 mW/m^2^, whereas the PD of the composite GO–PANI–Ag anode was 2.09 mW/m^2^. The composite anode delivered twice as much energy efficiency as the unmodified GO as well as improved electron transport. Added to that, GO removed Cd(II) at a rate of 64% and Pb(II) at a rate of 57.52%(II), whereas the GO–PANI–Ag anode removed Cd(II) at a rate of 80.25% and Pb(II) at a rate of 78.10%. During the optimization of parameters, we found that a neutral pH environment was the best for the operation of MFCs, for pH directly affected bacterial activity by either improving or hindering the efficiency of MFCs.

Even though the fabricated electrodes worked well in terms of increasing electron transport, the organic substrate used in the MFCs exhibited less stability. During the optimization of the organic substrate, oil palm trunk sap achieved high voltage as well as high removal efficiency due to its stability in relation to bacterial species. The less stable organic substrate affected the removal efficiency, however, because the organic substrate failed to give enough power to the bacterial species and thus resulted in poor MFC performance. The long-term stability of organic substrates in MFCs for industrial scale use should thus be the focus of future research. Several additional sources of carbohydrate-based waste, including local Malaysian fruit waste, are readily available and could be studied in MFCs.

## Figures and Tables

**Table 1:** Summary of the physicochemical analysis of wastewater before and after treatment with metals.

Physicochemical Properties	Wastewater before Metal Treatment	Wastewater after Metal Treatment
pH	6.96	6.44
Odor	Unpleasant smell	Unpleasant smell
Temperature	26 ± 2 °C	26 ± 2 °C
Color	Dark yellowish	Dark yellowish
Electrical conductivity	65 μS/cm	80 μS/cm
Pb (II)	0 ppm	100 ppm
Cd (II)	0 ppm	100 ppm

**Table 2:** The anodes’ efficiency in removing metal ions via MFCs.

Electrodes		Supplemented Metals	Inoculation Source	Initial Concentration (ppm)	Operational Time (Days)	Remediation Efficiency (%)
Fabricated Anode	Cathode					
GO	Graphite rod	Pb (II)	Synthetic wastewater	100	0	0
					5	15.20
					10	24.01
					15	34.50
					20	44.00
					25	51.00
					30	55.00
					35	57.52
GO/PANI/Ag	Graphite rod				0	0
					5	15.30
					10	31.45
					15	43.90
					20	56.88
					25	69.00
					30	76.32
					35	78.10
GO	Graphite rod	Cd (II)	Synthetic wastewater	100	0	0
					5	9
					10	19.00
					15	32.10
					20	46.51
					25	59.32
					30	61.00
					35	64.00
GO/PANI/Ag	Graphite rod				0	0
					5	20.30
					10	32.90
					15	49.40
					20	63.50
					25	74.13
					30	76.90
					35	80.25

**Table 3:** List of isolated identified bacterial species from the GO and GO–PANI–Ag anodes.

Accession Number1 (6S rRNA Gene)	Bacterial Species	Query Cover (%)	Identity (%)
GO-anode biofilm bacterial species			
NR_042288.1	*Leucobacter aridicollis strain L-9*	98	97.8
NR_114929.1	*Leucobacter komagatae strain IFO 15245*	97	96.70
NR_024674.1	*Leucobacter albus strain IAM 14851*	96	94.70
NR_044932.1	*Microbacterium arabinogalactanolyticum strain DSM 8611*	96	94.81
NR_108849.1	*Pseudarthrobacter siccitolerans strain 4J27*	96	90.71
NR_145906.1	*Leucobacter zeae strain CC-MF41*	96	90.58
NR_074608.1	*Glutamicibacter arilaitensis Re117*	96	90.45
NR_104498.1	*Citricoccus parietis strain 02-Je-010*	95	91.80
GO/PANI/Ag anode biofilm bacterial species			
NR_042414.1	*Leucobacter iarius strain 40*	96	98
NR_112021.1	*Leucobacter komagatae strain IFO 15245*	96	97.8
NR_116649.1	*Micrococcus terreus strain V3M1*	93.17	96
NR_117546.1	*Citricoccus nitrophenolicus strain PNP1*	96	92
NR_042425.1	*Leucobacter luti strain RF6*	96	92.45
NR_024783.1	*Arthrobacter russicus strain A1-3*	96	90.81
NR_074770.2	*Pseudarthrobacter phenanthrenivorans Sphe3*	96	90.70
NR_104839.1	*Curtobacterium oceanosedimentum strain ATCC 31317*	96	91.61
NR_026159.1	*Rathayibacter rathayi strain DSM 7485*	96	92.52
NR_133713.1	*Canibacter oris strain DSM 27064*	96	92.21

**Table 4:** Effect of different organic substrates on the operation of MFCs.

Anode Electrode	Organic Substrate	Voltage (mV)	Measurement Duration (Days)	Removal Efficiency (%) of Pb(II)	Removal Efficiency (%) of Cd (II)
GO	Sugar cane juice	48	10	24.01	19.00
GO/PANI/Ag composite	Sugar cane juice	100	10	31.45	32.90
GO	Glucose	45	10	18.50	12.10
GO/PANI/Ag composite	Glucose	85	10	24.00	29.25
GO	Oil palm trunk sap	55	10	26.00	25.50
GO/PANI/Ag composite	Oil palm trunk sap	115	10	33.85	36.40

## Data Availability

The data that support the findings of this study are available from the corresponding/first author upon reasonable request.
